# Tracheal Stenosis Associated With Operation for Pneumothorax With Marfan Syndrome: A Case Report

**DOI:** 10.7759/cureus.67492

**Published:** 2024-08-22

**Authors:** Yosuke Hamada, Yoshinobu Ichiki, Hirozo Sakaguchi, Hiroyuki Nitanda, Hironori Ishida

**Affiliations:** 1 Department of Thoracic Surgery, Respiratory Center, Toranomon Hospital, Tokyo, JPN; 2 Department of General Thoracic Surgery, Saitama Medical University International Medical Center, Saitama, JPN

**Keywords:** case report, tracheomalacia, emergent intubation, tracheal stenosis, marfan syndrome

## Abstract

Marfan syndrome is a genetic disorder in which impaired protein leads to connective tissue weakness. We herein report a case of unexpected tracheal stenosis that was diagnosed just before an operation for a recurrent right pneumothorax with Marfan syndrome.

A 16-year-old boy with bilateral repeated pneumothoraces associated with Marfan syndrome came to our emergency room complaining of dyspnea. A chest radiograph showed recurrent right pneumothorax. An operation was planned due to prolonged air leakage even after chest tube drainage. On induction of general anesthesia for repairing pneumothorax, a sudden difficulty occurred during manual ventilation, and the blood oxygen saturation temporarily decreased to 50%. Therefore, emergent intubation with a single-lumen tube was applied, which led back to full saturation. Bronchoscopy revealed a tortuous and flattened trachea. An endobronchial blocker tube was applied due to difficulty in double-lumen tube insertion, and bullectomy was accomplished without any other unexpected events.

Patients with Marfan syndrome may have asymptomatic tracheal stenosis due to structural abnormalities and latent tracheomalacia, and general anesthesia could be a trigger to develop the symptoms. Surgeons should bear this in mind, cooperate with anesthesiologists well, and prepare for emergent intubation when managing patients with Marfan syndrome in the perioperative settings.

## Introduction

Impairment of glycoprotein fibrillin 1 (encoded by gene mutations with an autosomal dominant pattern of inheritance) is known to weaken connective tissue, which in turn triggers various clinical symptoms in Marfan syndrome. There is a sporadic type of this condition, and approximately one in four patients with Marfan syndrome have a de novo mutation [1]. Weak connective tissue forms weak cartilage of the trachea, which could lead to tracheomalacia [2]. The development of tracheomalacia during an operation due to direct structural pressure toward the trachea has been reported [3]. We herein report a case in which a patient with Marfan syndrome unexpectedly developed symptomatic tracheal stenosis probably due to structural abnormalities and latent tracheomalacia immediately after the induction of general anesthesia.

## Case presentation

**Figure 1:**
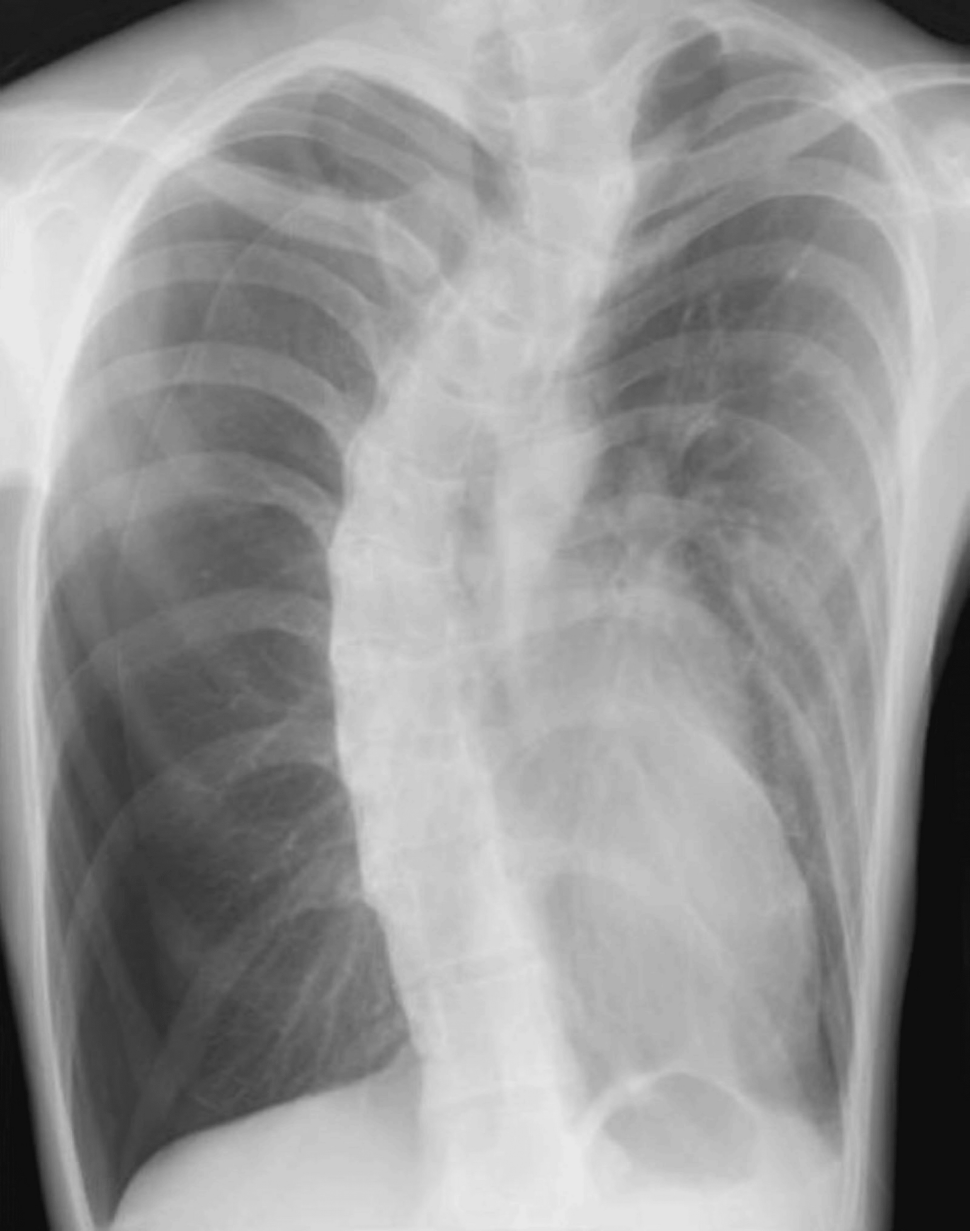
Preoperative chest radiograph. Right pneumothorax was developed. Scoliosis was significant.

The patient was a 16-year-old boy with dyspnea and right chest pain who had been diagnosed with Marfan syndrome based on a genetic test. There was no relevant family history. The patient had undergone bilateral repeated pneumothorax surgeries. A chest radiograph in the emergency room showed recurrent right pneumothorax (Figure 1).

A trocar catheter was inserted, and he was admitted for observation. Then, the third operation for the right pneumothorax was performed due to prolonged air leakage.

**Figure 2:**
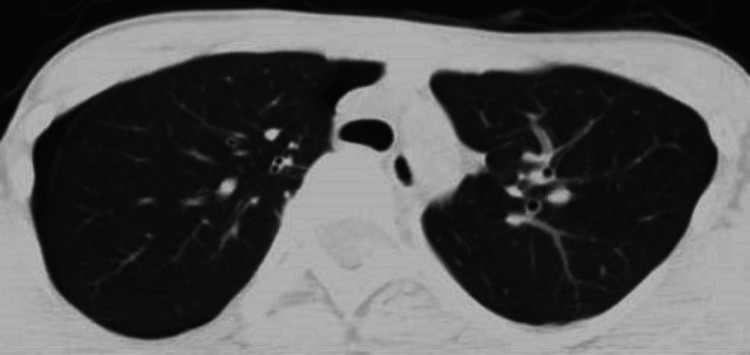
Preoperative computed tomography. Pectus excavatum and curved spine due to scoliosis seemed to have compressed the airway to some extent.

After the venous injection of sedative and muscle relaxant, the anesthesiologist experienced sudden difficulty with bag valve mask ventilation, and the blood oxygen saturation dropped to 50%. Emergent intubation with a single-lumen tube was applied, and the saturation returned to 100%. The preoperative computed tomography (CT) showed a slight narrowing of the trachea anteroposteriorly but no complete obstruction (Figure 2).

**Figure 3:**
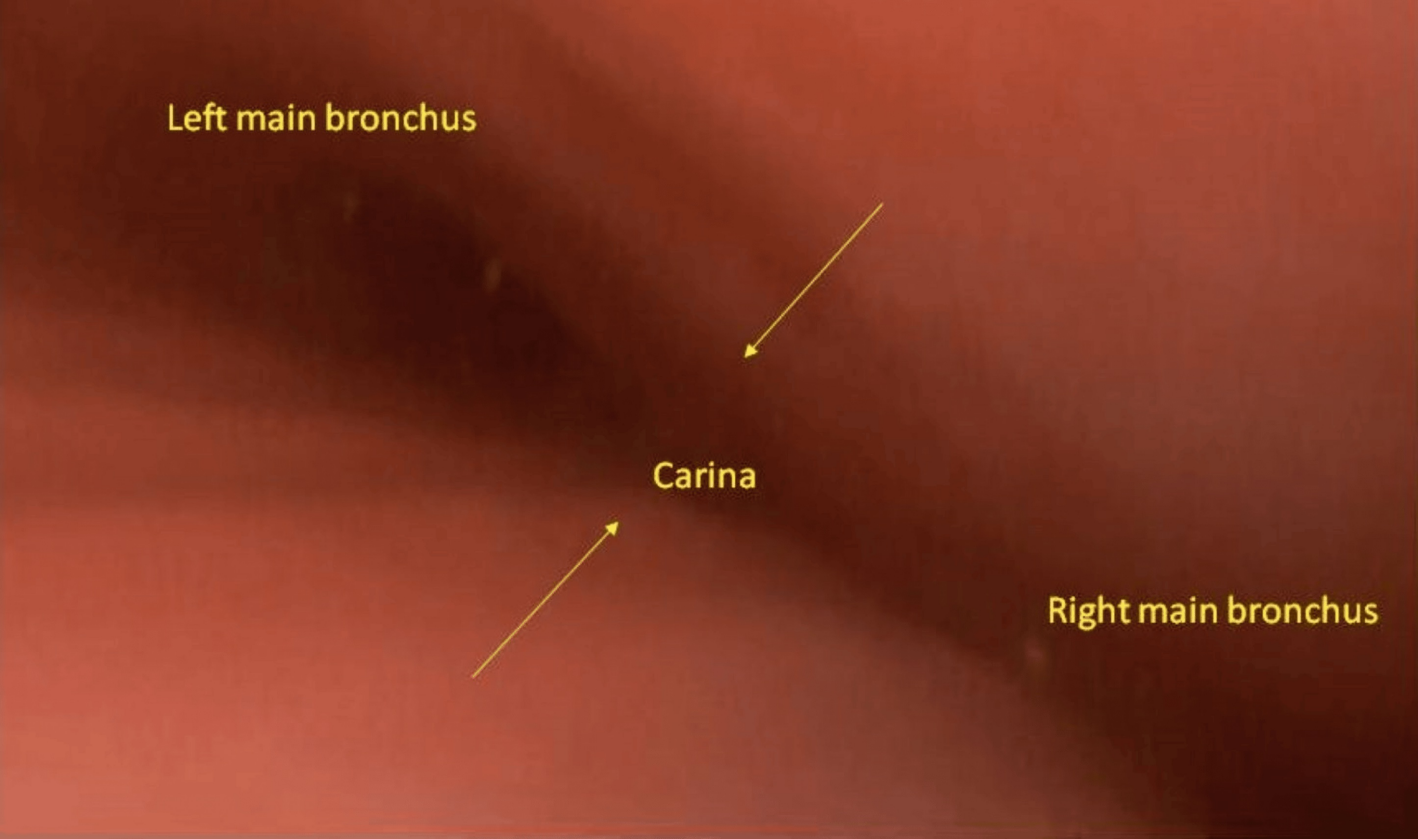
Bronchoscopic findings. The trachea was flattened anteroposteriorly and crescent-type tracheomalacia was observed.

The bronchoscopy after the induction of general anesthesia revealed a tortuous and flattened trachea and crescent-type tracheomalacia (Figure 3).

Several unsuccessful attempts were made to switch to a double-lumen tube. Therefore, a blocker with a single-lumen tube was used for differential lung ventilation.

**Figure 4:**
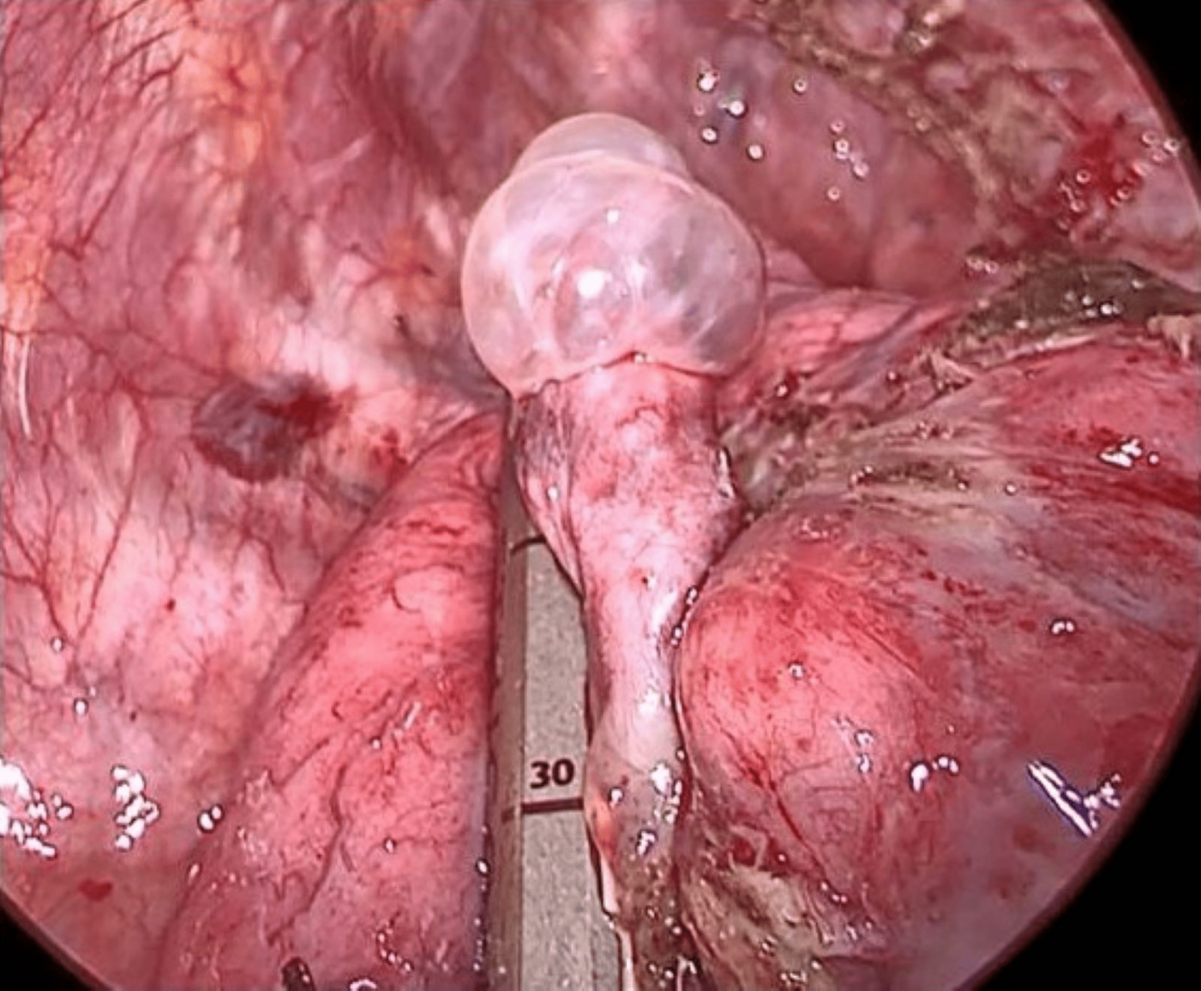
Intraoperative image of the bulla. The apical bulla was resected with a surgical stapler.

Intraoperatively, a 2-cm apical bulla was observed. A sealing test to check whether there was air leakage from the bulla with a positive pressure of 25 cm H2O was negative. The adhesion around the bulla was dissected, and bullectomy was performed with a surgical stapler (Figure 4).

**Figure 5:**
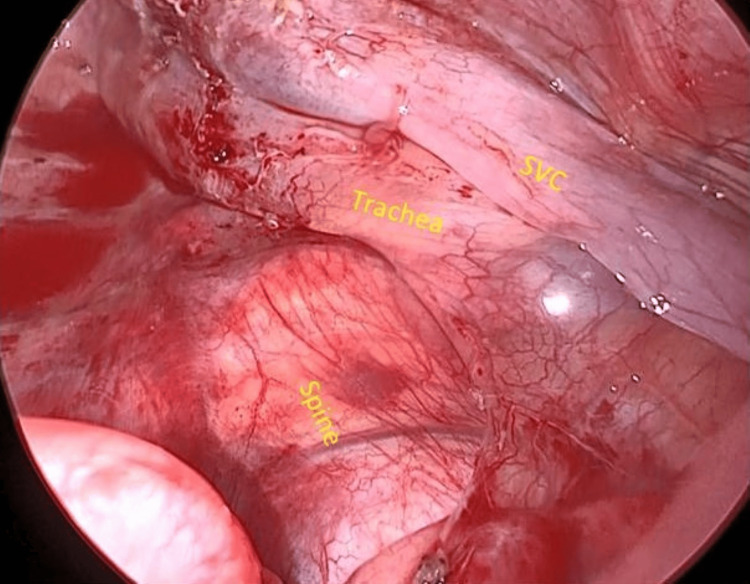
Intraoperative thoracoscopic findings. The curved spine seems to have compressed the trachea to some extent. SVC: superior vena cava.

Curved spine due to scoliosis, superior vena cava, and trachea were observed after the bulla was resected (Figure 5).

**Figure 6:**
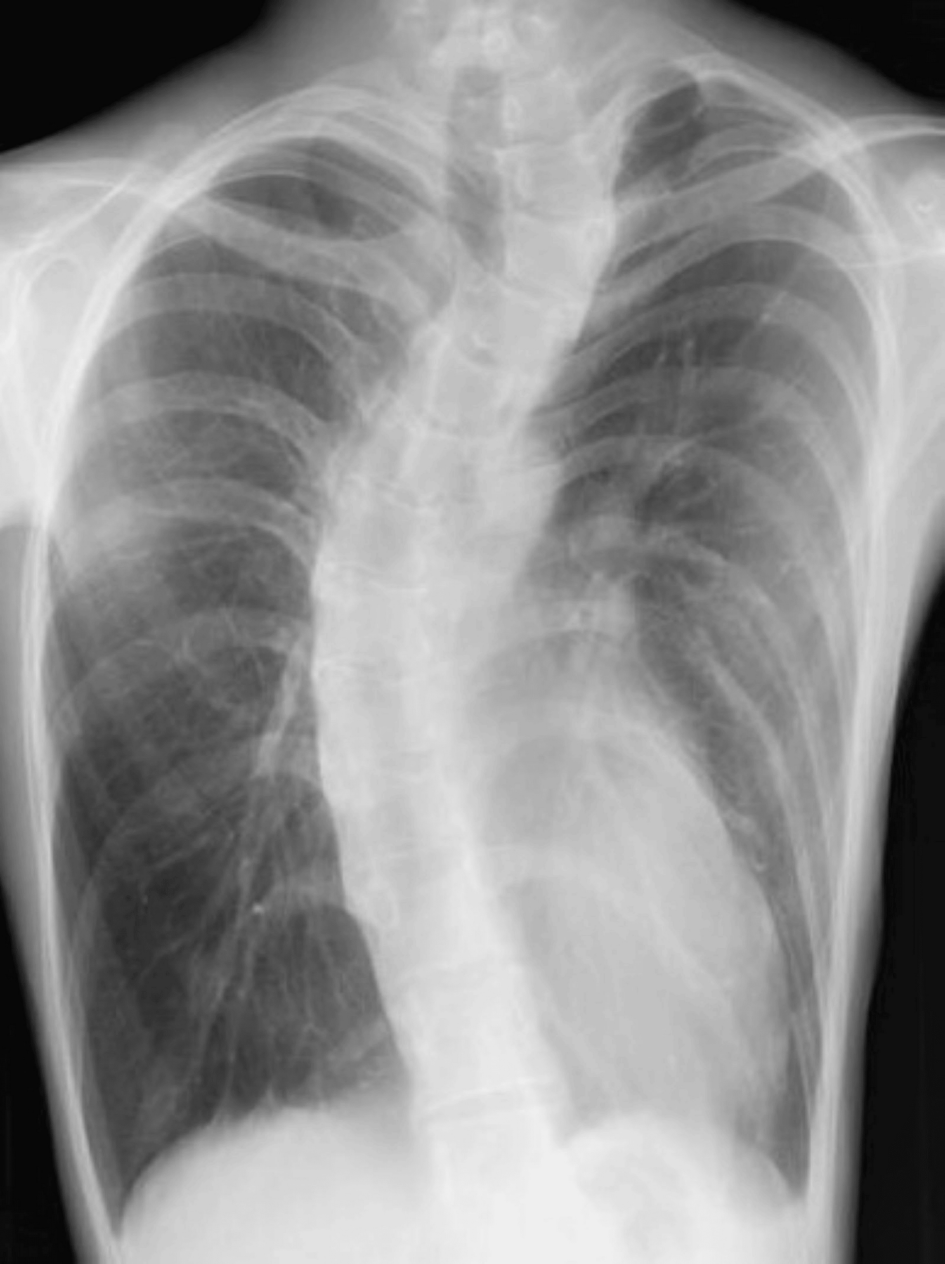
Postoperative chest radiograph. Full expansion of the right lung was obtained.

It seemed that there was a direct pressure from the spine toward the trachea to some extent. Extubation was carried out without any respiratory trouble after the anesthesia was discontinued although we were concerned about the risk of suffocation. The postoperative course was uneventful except for transient right lower lobe atelectasis. A chest radiograph at two months postoperatively showed complete expansion of the right lung (Figure 6).

**Figure 7:**
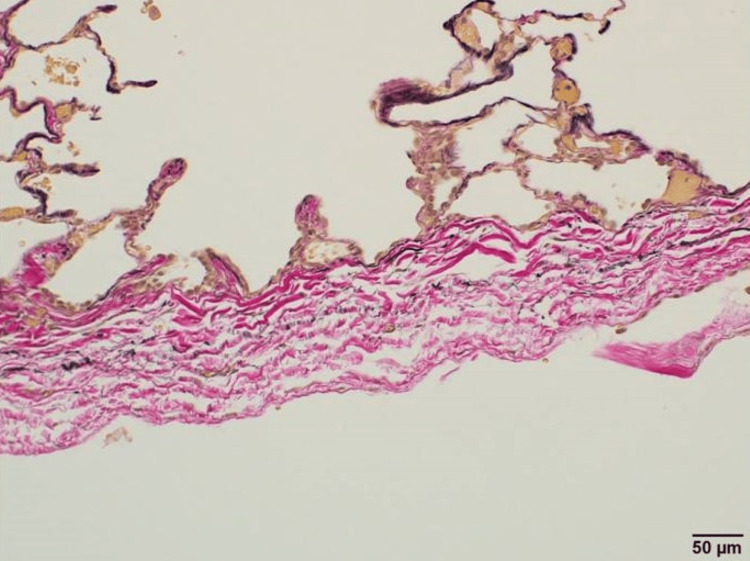
Pathological findings. Elastica van Gieson stain showed a decreased number of elastic fibers in the resected bulla.

In pathological findings of the resected bulla, the decreased number of elastic fibers was seen with the Elastica van Gieson stain (Figure 7).

## Discussion

Through this case with an important forewarning, we should know that dynamic tracheal stenosis can suddenly become symptomatic, provoked by general anesthesia [4]. Intrathoracic pressure is usually lower than airway pressure, but with bag valve mask ventilation, the intrathoracic and alveolar pressures markedly increase. Airway pressure decreases toward the thoracic outlet. When the supportability of the trachea is reduced due to its weak connective tissue, the trachea is easily constricted by external pressure, and tracheal stenosis is likely to occur especially during expiration [3,5]. The bronchoscopy revealed crescent-type tracheomalacia with anteroposterior obstruction, which is thought to be caused by weakening of the anterior cartilaginous wall [3,6].

Besides, patients with Marfan syndrome have been reported to have a decreased number of elastic fibers in resected bullae (Figure 7), which are degenerated and fragmented [7]. With this genetic condition, there might be a reduction of elastic fibers in the membranous part of the trachea as well, which together with the weak anterior cartilage led to this latent tracheomalacia, hence tracheal stenosis. Preoperative bronchoscopy may be considered for understanding the winding airway; however, the procedure without general anesthesia might not lead to the same findings as seen in this case.

Structural abnormalities may sometimes cause difficulty in ventilation. The preoperative CT showed no cardiovascular anomalies or mediastinal lesions compressing the trachea. However, there was a sign of mild compression of the trachea by the spine and the sternum both on the CT (Figure 2) and the intraoperative image (Figure 5). Hence, pectus excavatum and scoliosis may also have been responsible for the symptom in the present case. It is learned from this case that the risk of similar suffocation does exist with a narrowed airway on a preoperative CT and therefore you should be ready to deal with it even if a patient is asymptomatic before operation. When ventilation with a bag valve mask cannot be done, it can be usually resolved by emergent intubation and advancing the tube just above the carina.

## Conclusions

This case highlights the critical importance of recognizing the potential for tracheal stenosis to become symptomatic suddenly, particularly under general anesthesia. Structural abnormalities of Marfan syndrome, such as pectus excavatum and scoliosis, and possible tracheomalacia might contribute to tracheal stenosis. Preoperative bronchoscopy might be useful for understanding the airway's condition, though findings without general anesthesia might differ. The case underscores the importance of being prepared for airway management challenges, even in asymptomatic patients, as emergent intubation may be necessary when bag valve mask ventilation fails. If similar problems occur, we should intubate the patient without hesitation.
